# Quantum Otto Heat Engine Using Polar Molecules in Pendular States

**DOI:** 10.3390/molecules29235617

**Published:** 2024-11-27

**Authors:** Xiang Li, Zhaoxi Sun, Yu-Yan Fang, Xiao-Li Huang, Xinning Huang, Jin-Fang Li, Zuo-Yuan Zhang, Jin-Ming Liu

**Affiliations:** 1 College of Physical Science and Technology, Yangzhou University, Yangzhou 225009, China; 2 Changping Laboratory, Beijing 102206, China; 3 State Key Laboratory of Precision Spectroscopy, School of Physics and Electronic Science, East China Normal University, Shanghai 200241, China; 4 Department of Physics, Liaoning Normal University, Dalian 116029, China; 5 Department of Physics and Electronic Engineering, Xianyang Normal University, Xianyang 712000, China; 6 Chongqing Key Laboratory of Precision Optics, Chongqing Institute of East China Normal University, Chongqing 401120, China

**Keywords:** polar molecules, quantum heat engine, quantum entanglement

## Abstract

Quantum heat engines (QHEs) are established by applying the principles of quantum thermodynamics to small−scale systems, which leverage quantum effects to gain certain advantages. In this study, we investigate the quantum Otto cycle by employing the dipole−dipole coupled polar molecules as the working substance of QHE. Here, the molecules are considered to be trapped within an optical lattice and located in an external electric field. We analyze the work output and the efficiency of the quantum Otto heat engine (QOHE) as a function of various physical parameters, including electric field strength, dipole−dipole interaction and temperatures of heat baths. It is found that by adjusting these physical parameters the performance of the QOHE can be optimized effectively. Moreover, we also examine the influences of the entanglement and relative entropy of coherence for the polar molecules in thermal equilibrium states on the QOHE. Our results demonstrate the potential of polar molecules in achieving QHEs.

## 1. Introduction

Thermodynamics was initially developed to improve the performance of large−scale machinery; however, it has been extended to the quantum scale in recent years, leading to the emergence of quantum thermodynamics [1,2,3]. As a key topic in quantum thermodynamics, quantum heat engines (QHEs) have attracted widespread attention due to their potential applications in energy production, transportation, and computation [4,5]. The distinguishing feature of QHEs is that they use quantum systems as the working medium, such as quantum dots [6,7], trapped ions [8], atomic ensembles [9,10,11], optomechanical oscillators [12,13], nuclear magnetic resonance setup [14], etc. This enables the exploitation of quantum advantages including superposition and coherence [15,16,17]. Consequently, the QHEs may outperform the equivalent classical heat engines in certain aspects [18,19,20]. As the quantum counterpart of classical combustion engines, the quantum Otto heat engine (QOHE) follows a similar principle with two isochoric and two adiabatic steps, has been widely investigated in recent years. For example, Camati et al. [16] analyzed the role of coherence in the QOHE, establishing a direct correlation between power and efficiency with energy coherence. Gao et al. [21] demonstrated that the maximum power of the coupled−qubit QOHE is greater than that of the single−qubit QOHE. Moreover, in Heisenberg spin system, the influences of different physical parameters (e.g., magnetic field and Dzyaloshinskii−Moriya interaction) on the quantum Otto cycle were studied in Refs. [5,22]. Lately, the experimental realizations of the QOHEs in different quantum systems have been reported [8,9,14,23].

Polar molecules have the coherence times comparable to those of neutral atoms, strong controllable long−range interactions, and rich internal degrees of freedom, playing a key role in quantum computation [24,25,26,27,28], quantum simulation [29,30], precision measurement [31], and controlled chemistry [32]. In 2002, the proposal for a prototype of a polar molecule-based quantum computer was put forward by DeMille [24]. In DeMille’s proposal, the electric dipole moments of lattice−confined polar molecules, which are oriented along or against an external electric field, can serve as qubits. These qubits are coupled through electric dipole−dipole interaction, and could be used to achieve approximately $10^{5}$ CNOT gates based on ultracold KCs molecules. Inspired by [24], we designed the optimal control pulses that interact with the polar YbF molecules by employing the recently popular machine learning technique [28]. Then, the NOT, CNOT, and Hadamard gates were implemented with high fidelities. Moreover, in recent years, the quantum correlation and coherence for polar molecules have been extensively investigated [33,34,35,36,37]. For example, Wei et al. [33] studied the dependence of entanglement in polar molecules on the strength of the external electric field, dipole−dipole coupling, and ambient temperature. Recently, we have successfully demonstrated the generation of entanglement in polar molecular systems through the utilization of deep reinforcement learning [37]. On the other hand, significant advancements have been made in diverse methodologies and technologies related to the cooling, trapping, and manipulation of cold molecules over the past few decades [38,39,40,41], establishing polar molecules as a highly promising candidate for quantum information processing.

Note that, in some existing studies, the molecules have been considered as a platform for implementing QHEs. For example, Hübner et al. [42] presented the molecular quantum Otto cycle utilizing the Ni_2_ dimer in the presence of a static magnetic field and discussed the relationship between efficiency and entanglement. It is found that the performance of the cycles is significantly affected by the detailed electronic energy levels. Moreover, Chen et al. [43] considered a generic donor−bridge−acceptor molecular model, and investigated the effects of quantum coherence and dephasing on the performance of molecular heat engines by utilizing nonequilibirum Green function methodology.

In this paper, we consider a qubit−qubit system consisting of two polar molecules coupled by the dipole−dipole interaction as the working substance of the QOHE. The molecules are assumed to be located in a static electric field, resulting in the superposition of molecular rotational modes into pendular qubit states. Our results show that the work output and efficiency of the QOHE can be manipulated by adjusting the electric field strength, the dipole−dipole interaction, and the ambient temperature. Moreover, the effects of thermal entanglement and coherence on the performance of QOHE are discussed. Our results could shed some light on the implementation of QHEs based on polar molecules.

The outline of this paper is organized as follows. In Section 2, we briefly review the model of polar molecules in pendular states and the quantum Otto cycle. In Section 3, the evolution behaviors of the work output and efficiency for the QOHE are numerically analyzed. We summarize our conclusions in Section 4.

## 2. Theory

### 2.1. Polar Molecules in Pendular States

**Figure 1:**
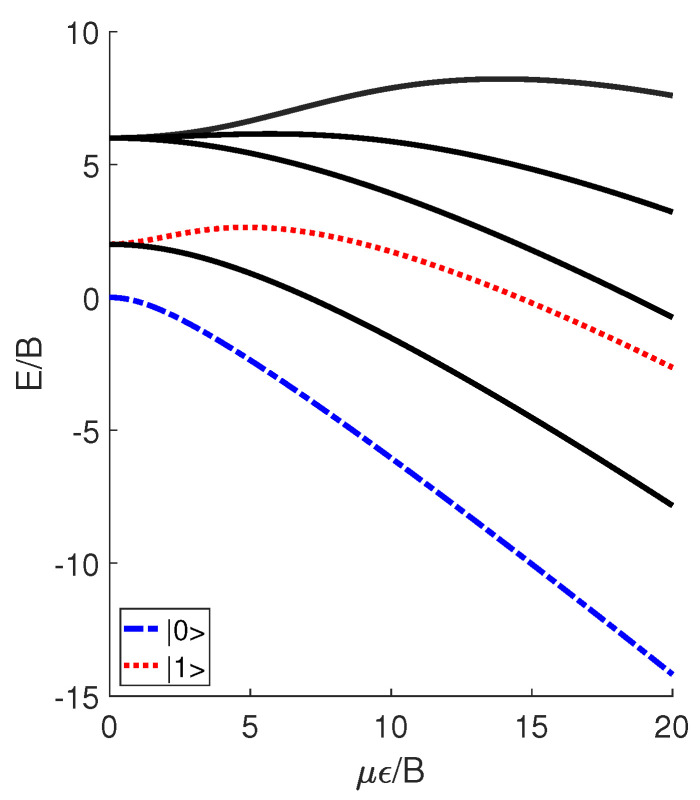
Several unitless reduced pendular levels E/B of an ultracold polar molecule as a function of μϵ/B. Here, qubits $|0\rangle$ and $|1\rangle$ are encoded in the levels for M=0.

The Hamiltonian of a polar diatomic or linear molecule in the presence of an electric field can be expressed by [33]

(1) $$H=BJ^{2}-\mu \epsilon \cos\theta ,$$

where *B* denotes the molecular rotational constant, *J* denotes the angular momentum operator, and θ denotes the angle between the molecular permanent dipole moment μ and the electric field ϵ. The introduction of the external electric field leads to the generation of pendular states, which are actually the superposition of molecular rotational modes. In Figure 1, we plot the unitless energy levels E/B of the pendular states versus the unitless parameter μϵ/B, where *E* denotes the eigenenergies of the Hamiltonian *H*. Note that, in this study, all of the results are presented using the unitless reduced variables, ensuring that the conclusions obtained are applicable to various polar diatomic molecules rather than just a specific one. DeMille [24] utilized the two lowest pendular states with M=0 to define qubits $|0\rangle$ and $|1\rangle$, which can be expressed as linear combinations of spherical harmonics $Y_{j,0}\left(\theta ,\phi \right)$ and symbolized by

(2) $$|0\rangle =\sum_{j}a_{j}Y_{j,0}\left(\theta ,\phi \right),\;|1\rangle =\sum_{j}b_{j}Y_{j,0}\left(\theta ,\phi \right),$$

where ϕ spans from 0 to 2π, and $a_{j}$ and $b_{j}$ are the coefficients of the sum of the spherical harmonics. Under the pendular qubit states |0〉 and |1〉, the effective dipole moments of the polar molecule are described as $C_{0}=\langle 0\left|\cos\theta \right|0\rangle$ and $C_{1}=\langle 1\left|\cos\theta \right|1\rangle$, and the transition dipole moment between the two pendular states can be expressed as $C_{t}=\langle 0\left|\cos\theta \right|1\rangle$.

When considering another identical polar molecule in the same static electric field, the dipole−dipole interaction between the two coupled molecules for M=0 can be simplified as [28,33]

(3) $$V_{d-d}=\Omega \left(1-3\cos^{2}\alpha \right)\cos\theta_{1}\cos\theta_{2},$$

where $\Omega =\mu^{2}/r_{12}^{3}$ can be regarded as the coupling strength, $r_{12}=\left|r_{1}-r_{2}\right|$ is the intermolecular distance, and α is the angle between $r_{12}$ and the electric field’s direction. As a result, the total Hamiltonian of the dipole−dipole coupled molecular system in an electronic field is given by

(4) $$H^{\prime }=H_{1}+H_{2}+V_{d-d}.$$

Under the basis of the pendular qubits $\left\{|00\rangle ,|01\rangle ,|10\rangle ,|11\rangle \right\}$, the Hamiltonian $H_{1}$ is expanded as

$$H_{1}=\left(\begin{array}{cc} E_{0}^{1} & 0\\ 0 & E_{1}^{1} \end{array}\right)⊗I,$$

where *I* denotes a 2×2 identity matrix, $E_{0}^{1}$ and $E_{1}^{1}$ are the eigenenergies versus the pendular states $|0\rangle$ and $|1\rangle$ for the first molecule. Similarly, the Hamiltonian $H_{2}$ is

$$H_{2}=I⊗\left(\begin{array}{cc} E_{0}^{2} & 0\\ 0 & E_{1}^{2} \end{array}\right),$$

where $E_{0}^{2}$ and $E_{1}^{2}$ are the eigenenergies versus the pendular states $|0\rangle$ and $|1\rangle$ for the second molecule. Additionally, the dipole−dipole interaction term $V_{d-d}$ is given by

(5) $$V_{d-d}=\Omega \left(\begin{array}{cc} C_{0}^{1} & C_{t}^{1}\\ C_{t}^{1} & C_{1}^{1} \end{array}\right)⊗\left(\begin{array}{cc} C_{0}^{2} & C_{t}^{2}\\ C_{t}^{2} & C_{1}^{2} \end{array}\right).$$

Here, $C_{0}^{x}=\langle 0\left|\cos\theta \right|0\rangle$ and $C_{1}^{x}=\langle 1\left|\cos\theta \right|1\rangle$ (x=1,2) characterize the effective dipole moments for the *x*th polar molecule, while $C_{t}^{x}=\langle 0\left|\cos\theta \right|1\rangle$ characterizes the transition dipole moment.

To explore the thermal properties of the QOHE using the coupled polar molecules in an electric fields as the working substance, we consider the thermal equilibrium state of the molecular system at temperature *T*, for which the density matrix can be written as

(6) $$\rho \left(T\right)=\frac{1}{Z(T)}\overset{4}{\sum_{i=1}}e^{-\frac{1}{k_{B}T}E_{i}}|\psi_{i}\rangle \langle \psi_{i}|.$$

Here, $|\psi_{i}\rangle$ represents the *i*th eigenstate corresponding to the eigenvalue $E_{i}$ of the total Hamiltonian $H^{\prime }$, $k_{B}$ denotes Boltzmann’s constant, and Z(T) is the partition function with the following form

(7) $$Z\left(T\right)=\mathrm{Tr}\left[\exp\left(-\frac{H^{\prime }}{k_{B}T}\right)\right],$$

where the trace operation Tr sums over all exponentiated eigenvalues of the Hamiltonian.

We note that, in 2018, the CaF molecule has been laser cooled to a temperature regime of microkelvin [44]. Recently, the entanglement of CaF molecules in an optical tweezer array has been successfully prepared in experiments [45,46]. These achievements may make the CaF molecule a suitable test carrier for QHEs.

### 2.2. Quantum Otto Cycle

The quantum Otto cycle of coupled bipolar molecules as working substances consists of two quantum isochoric processes and two quantum adiabatic processes [3], which can be described in four distinct stages.

**Stage One: Quantum isochoric heating.** In this process, the two polar molecules, which are subjected to an electric field $\epsilon_{h}$, serve as the working substance and come into contact with a hot heat bath at temperature $T_{h}$. Here, the dipole−dipole coupling strength between the two molecules is denoted as $\Omega_{h}$. After a sufficient period, the molecular system reaches thermal equilibrium with the heat source. The density matrix of the working substance at this point is defined as $\rho_{h}=\exp\left(-H_{h}^{\prime }/k_{B}T_{h}\right)/Z_{h}=\sum_{n}p_{n}^{h}|\Psi_{n}\rangle \langle \Psi_{n}|$, where $H_{h}^{\prime }$ represents the system’s Hamiltonian at this stage, $p_{n}^{h}=\exp\left(-E_{n}^{h}/k_{B}T_{h}\right)/Z_{h}$ denotes the population probabilities for each eigenstate, and $Z_{h}=\sum_{n}\exp\left(-E_{n}^{h}/k_{B}T_{h}\right)$ is the partition function. During this process, heat $Q_{h}$ is transferred from the heat source to the working substance.

**Stage Two: Quantum adiabatic expansion.** During this stage, the system is isolated from the heat source and undergoes an adiabatic expansion. This stage must be executed slowly enough to satisfy the quantum adiabatic theorem, ensuring that the population probabilities $p_{n}^{h}$ of each eigenstate remain unchanged. Note that, in this stage, there is no heat exchange between the system and the environment, and the system’s Hamiltonian transforms to $H_{c}^{\prime }$ from $H_{h}^{\prime }$ at the end of evolution.

**Stage Three: Quantum isochoric cooling.** In this stage, the working substance is brought into contact with a cold bath at $T_{c}$, and undergoes another quantum isochoric process, where the electric field and the dipole−dipole coupling strength are represented by $\epsilon_{c}$ and $\Omega_{c}$, respectively. At the conclusion of this stage, the system and the low-temperature heat source reach thermal equilibrium. Then, the density matrix is changed to $\rho_{c}=\exp\left(-H_{c}^{\prime }/k_{B}T_{c}\right)/Z_{c}=\sum_{n}p_{n}^{c}|\Psi_{n}\rangle \langle \Psi_{n}|$, where $p_{n}^{c}=\exp\left(-E_{n}^{c}/k_{B}T_{c}\right)/Z_{c}$, and $Z_{c}=\sum_{n}\exp\left(-E_{n}^{c}/k_{B}T_{c}\right)$. During the process of time evolution, a quantity of heat $Q_{c}$ is transferred from the working substance to the low−temperature heat source.

**Stage Four: Quantum adiabatic compression.** In this final stage, the external electric field recovers to $\epsilon_{h}$ from $\epsilon_{c}$, and the coupling strength recovers to $\Omega_{h}$ from $\Omega_{c}$. The probabilities $p_{n}^{c}$ remain unchanged. At the end of evolution, the system’s Hamiltonian reverts to $H_{h}^{\prime }$.

When the entire cycle is completed, the heat transferred can be quantitatively analyzed based on the quantum form of the first law of thermodynamics as follows [2,22]:

(8) $$Q_{h}=Tr\left(H_{h}^{\prime }\rho_{h}\right)-Tr\left(H_{h}^{\prime }\rho_{c}\right)=\sum_{n}E_{n}^{h}\left(p_{n}^{h}-p_{n}^{c}\right),$$

(9) $$Q_{c}=Tr\left(H_{c}^{\prime }\rho_{c}\right)-Tr\left(H_{c}^{\prime }\rho_{h}\right)=\sum_{n}E_{n}^{c}\left(p_{n}^{c}-p_{n}^{h}\right).$$

Here, $Q_{h}>0$ and $Q_{c}<0$ characterize the processes of the absorption and release of heat, respectively. The net work output *W* of the QOHE, which is determined by the principles of energy conservation, is given by

(10) $$W=Q_{h}+Q_{c}=\sum_{n}\left(E_{n}^{h}-E_{n}^{c}\right)\left(p_{n}^{h}-p_{n}^{c}\right).$$

When W>0, the engine performs positive work. As a result, the efficiency of the heat engine can be expressed as $\eta =W/Q_{h}$.

## 3. Results

### 3.1. QOHE of Coupled Dipoles in Thermal Equilibrium

**Figure 2:**
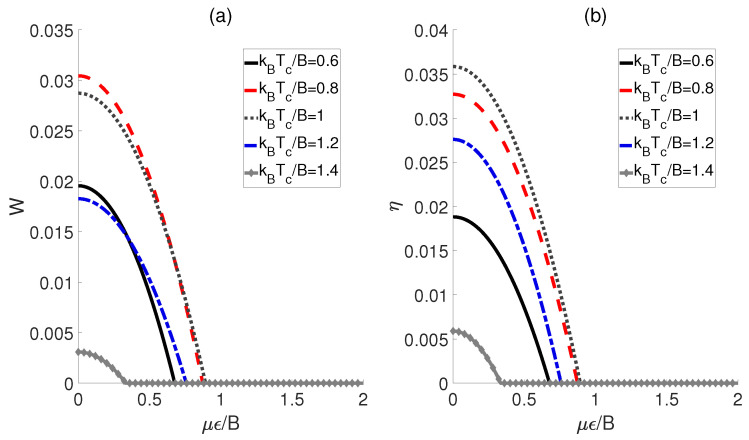
Work output *W* (**a**) and efficiency η (**b**) as functions of the external electric field strength μϵ/B and cold bath temperatures $k_{B}T_{c}/B$. Here, $k_{B}T_{h}/B=3$, $\Omega_{h}/B=5$, and $\Omega_{c}/\Omega_{h}=0.6$.

First, the influences of the external electric field and the temperatures $T_{c}$ of the cold bath on the performance of a QOHE based on polar molecules are explored. Here, we consider the scenario in which the dipole−dipole coupling strength Ω experiences a transformation of $\Omega_{h}\to \Omega_{c}\to \Omega_{h}$, while the external electric fields ϵ corresponding to the cold and hot baths remain identical (i.e., $\epsilon_{h}=\epsilon_{c}=\epsilon$). As shown in Figure 2, both the output work *W* and the efficiency η exhibit similar behaviors with respect to μϵ/B for different temperatures $T_{c}$. Concretely speaking, in Figure 2a, we can see that the output work *W* for each $k_{B}T_{c}/B$ initiates at a non−zero value, and then decreases monotonically to zero as μϵ/B increases. This means that within a limited electric field strength, the QOHE can work normally. However, beyond this range, the positive work cannot be output. Furthermore, we observe that the maximum work output of QOHE is achieved at $k_{B}T_{c}/B=0.8$ when μϵ/B is relatively weak. Moreover, the evolution behaviors of the heat engine efficiency η are plotted in Figure 2b. For a given μϵ/B, it can be found that the maximum value of η occurs at $k_{B}T_{c}/B=1$. However, $k_{B}T_{c}/B=1$ is not the minimum value among the several given $k_{B}T_{c}/B$ but rather a middle value, indicating that the excessive temperature differences between the cold and heat baths might reduce the output efficiency of the QOHE based on the polar molecules.

**Figure 3:**
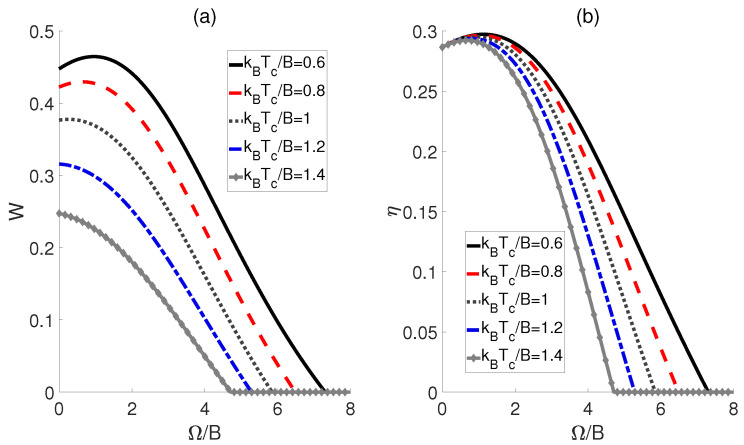
Work output *W* (**a**) and efficiency η (**b**) of the QOHE as functions of dipole−dipole coupling strength Ω/B and cold bath temperatures $k_{B}T_{c}/B$. Here, $k_{B}T_{h}/B=3$, $\epsilon_{h}=5$, and $\epsilon_{c}/\epsilon_{h}=0.6$.

Then, we investigate the scenario where the external electric field ϵ varies as the working substance comes into contact with the cold and hot baths, i.e., $\epsilon_{h}\to \epsilon_{c}\to \epsilon_{h}$, while maintaining identical dipole−dipole interactions, i.e., $\Omega_{h}=\Omega_{c}=\Omega$. In this setup, the impact of cold bath temperatures $T_{c}$ on the performance of QOHE is also considered. For $k_{B}T_{c}/B=0.6$, 0.8 and 1, Figure 3a demonstrates that the work output *W* initially increases and reaches a peak as Ω/B is elevated, but then declines with further increases in Ω/B. This implies the existence of an optimal coupling strength at which the maximum work output can be attained. However, it should be noted that when the temperature $T_{c}$ is high, e.g., $k_{B}T_{c}/B=1.2$ and 1.4, *W* will decrease monotonously with the increase in Ω/B. Figure 3b indicates that as Ω/B increases, the efficiency η for all $k_{B}T_{c}/B$ exhibits a single−peak trajectory. Thus, the maximum efficiency of the QOHE can be obtained by precisely controlling Ω/B. Furthermore, from Figure 3, we can observe that both the output work and efficiency for a given Ω/B always increase as the temperature difference $\Delta T=T_{h}-T_{c}$ expands, which contrasts with the case shown in Figure 2.

**Figure 4:**
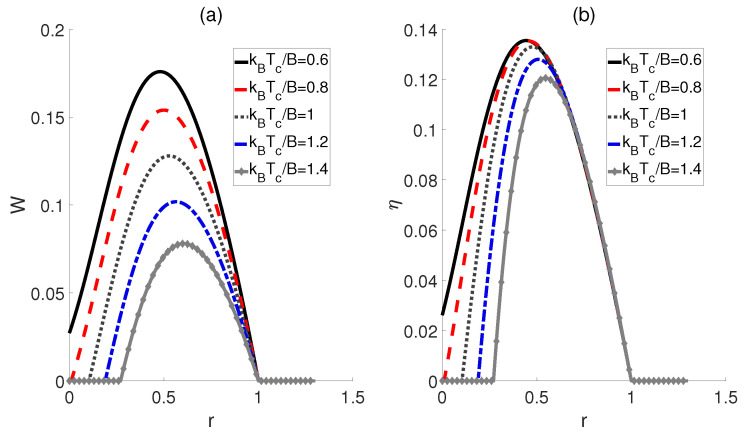
Impact of the ratio *r* on the work output *W* (**a**) and efficiency η (**b**) of the QOHE. Here, $k_{B}T_{h}/B=3$, $\mu \epsilon_{h}/B=3$, and $\Omega_{h}/B=3$.

Inspired by Ref. [22], we subsequently proposed an alternative scenario wherein the external electric fields and the dipole−dipole interaction are proportionally linked across adiabatic transitions, denoted as $\epsilon_{c}/\epsilon_{h}=\Omega_{c}/\Omega_{h}=r$, where *r* represents a ratio. In Figure 4, we plot the work output *W* and efficiency η as functions of the ratio *r*. The curves for different temperatures $T_{c}$ of the cold bath exhibit a peak followed by a subsequent decline as *r* increases, thereby confirming the existence of the optimal values of *r* that can improve the performance of the heat engine. Moreover, Figure 4b shows that the highest efficiency η is achieved at the lowest temperature $k_{B}T_{c}/B=0.6$ for a given ratio *r*, which is consistent with the prior experience in classical thermodynamic cycles, i.e., a larger temperature difference between the high− and low−temperature heat sources can result in higher efficiency. Furthermore, the peaks of these efficiency curves are found to shift rightward with decreasing temperature differences, indicating that smaller temperature differences require a higher ratio *r* to achieve maximum work output and efficiency. However, it is worth noting that as the ratio *r* increases to a critical value, the impact of temperature differences diminishes, leading to the convergence of multiple curves. If r>1, *W* and η for different temperatures $T_{c}$ tend to 0.

**Figure 5:**
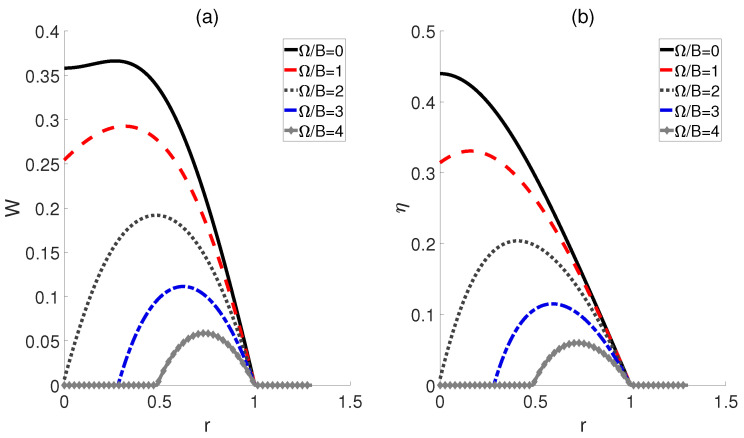
Work output *W* (**a**) and efficiency η (**b**) of a QOHE as functions of the ratio *r* = $\epsilon_{c}/\epsilon_{h}$ and coupling strength Ω/B. Here, $k_{B}T_{h}/B=3$, $k_{B}T_{c}/B=1$, and $\mu \epsilon_{h}/B=3$.

Finally, we consider the scenario where the electric fields corresponding to the hot and cold baths remain proportional, denoted as $\epsilon_{c}/\epsilon_{h}=r$, while the coupling strengths $\Omega_{c}$ and $\Omega_{h}$ are equal, denoted as $\Omega_{c}$ = $\Omega_{h}$ = Ω. Here, the temperatures for hot and cold baths are fixed. As shown in Figure 5, for a given ratio *r*, the smaller the Ω/B, the larger the work output *W* and the efficiency η become. Moreover, with an increase in Ω/B, the ratios *r* corresponding to the peaks of *W* and η exhibit a shift towards larger values. Additionally, it should be noted that when the ratio *r* exceeds 1, both *W* and η become 0 for all Ω/B, which is similar to the cases depicted in Figure 4.

### 3.2. Effects of Entanglement and Coherence on QOHE

In this subsection, we investigate how the thermal entanglement (characterized by concurrence) and coherence (quantified by relative entropy) influence the QOHE. For a qubit−qubit system, the concurrence *c* is defined as [47]:

(11) $$c=\max\{0,\sqrt{\lambda_{1}}-\sqrt{\lambda_{2}}-\sqrt{\lambda_{3}}-\sqrt{\lambda_{4}}\}.$$

Here, $\lambda_{i}$ with i=1,2,3,4 are the eigenvalues of the matrix *M*, listed in descending order, with

(12) $$M=\rho \left(\sigma_{y}⊗\sigma_{y}\right)\rho^{*}\left(\sigma_{y}⊗\sigma_{y}\right),$$

where ρ is the density matrix of an arbitrary two−qubit state, $\rho^{*}$ is the complex conjugate of ρ, and $\sigma_{y}$ represents the Pauli−Y operator. For maximally entangled states, c=1; for completely separable states, c=0; for partially entangled states, 1>c>0.

**Figure 6:**
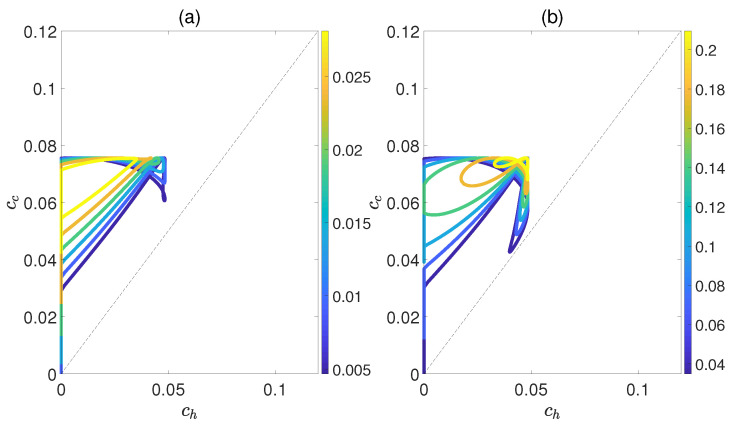
(Color online) Variations in work output *W* (**a**) and the efficiency η (**b**) with the concurrence $c_{h}$ and $c_{c}$ in isoline maps. Here, the relative coupling strength k=0.5, $k_{B}T_{c}/B=0.5$, and $T_{h}=2T_{c}$.

**Figure 7:**
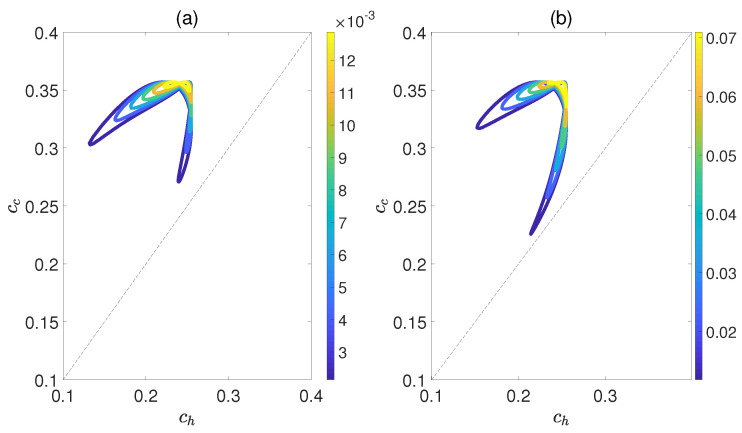
(Color online) Variations in work output *W* (**a**) and the efficiency η (**b**) with the concurrence $c_{h}$ and $c_{c}$ in isoline maps. Here, the relative coupling strength k=1.5, $k_{B}T_{c}/B=0.5$, and $T_{h}=2T_{c}$.

Figure 6 demonstrates the dependence of the work output *W* and the efficiency η on the concurrence $c_{h}$ and $c_{c}$, where $c_{h}$ and $c_{c}$ denote the entanglement for the state of the working substance reaching thermal equilibrium with the high− and low−temperature baths, respectively. Here, we introduce a relative coupling strength *k*, defined as the ratio between the dipole−dipole coupling strength and the coupling strength between dipole moment μ and electric field ϵ, i.e., k=Ω/μϵ. In Figure 6, *k* is set to 0.5. We can observe that both *W* and η exhibit non−monotonic behaviors with respect to $c_{h(c)}$, while keeping $c_{c(h)}$ fixed. This highlights an optimal entanglement range that enables the heat engine to work most efficiently. In addition, the density of contour lines varies within different ranges of $c_{c}$ and $c_{h}$. The denser the contour lines are, the more sensitive *W* and η become to entanglement, and vice versa. Furthermore, we also consider the case of k=1.5, as shown in Figure 7. The trends of *W* and η exhibit similarities to those depicted in Figure 6, except that their order of magnitude is smaller and the non−monotonicities versus $c_{c}$ and $c_{h}$ become more apparent. Moreover, regardless of whether *k* is 0.5 or 1.5, the QOHE can only generate net work within the range of $c_{h}<c_{c}$. If $c_{h}>c_{c}$, then W=η=0. Consequently, it can be inferred that the entanglement of polar molecules in thermal states significantly influences the performance of QOHE.

Quantum coherence is an essential physical resource in quantum information processing, which arises from the superposition of quantum states. Here, we utilize the relative entropy to evaluate the coherence of the molecular system in thermal equilibrium. In Ref. [48], the relative entropy of coherence is given by

(13) $$rec=S\left(\rho_{diag}\right)-S\left(\rho \right).$$

**Figure 8:**
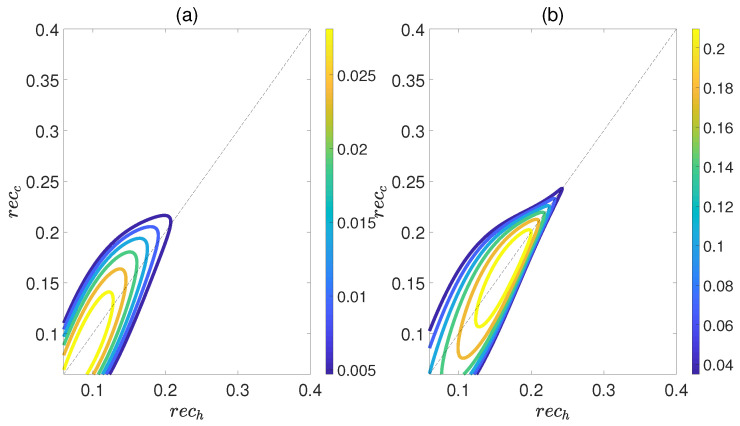
(Color online) Variations in the work output *W* (**a**) and the efficiency η (**b**) with the coherence $rec_{h}$ and $rec_{c}$ in isoline maps. Here, the relative coupling strength k=0.5, $k_{B}T_{c}/B=0.5$, and $T_{h}=2T_{c}$.

**Figure 9:**
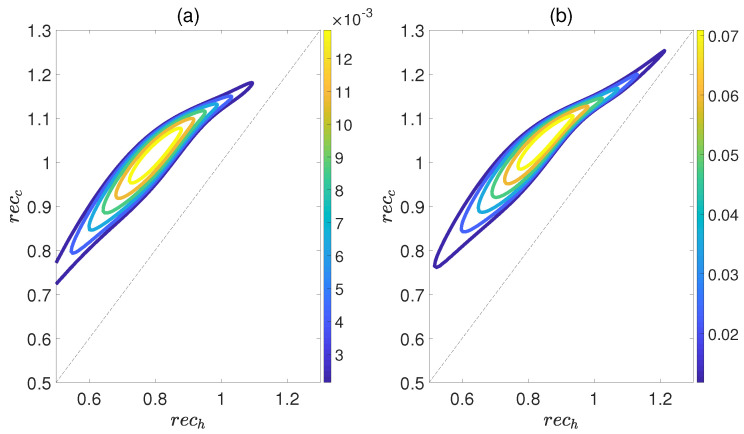
(Color online) Variations in the work output *W* (**a**) and the efficiency η (**b**) with the coherence $rec_{h}$ and $rec_{c}$ in isoline maps. Here, the relative coupling strength k=1.5, $k_{B}T_{c}/B=0.5$, and $T_{h}=2T_{c}$.

Here, $\rho_{diag}$ is the density matrix obtained by deleting all the off−diagonal elements of the quantum state ρ, and $S\left(\rho \right)=-\mathrm{Tr}\left(\rho \log_{2}\rho \right)$ is the von Neumann entropy. The work output *W* and efficiency η are depicted in Figure 8 as functions of $rec_{c}$ and $rec_{h}$, while maintaining a relative coupling strength of k=0.5. Here, $rec_{c}$ and $rec_{h}$ represent the coherence of polar molecules as they reach thermal equilibrium with the cold and hot baths, respectively. It is evident that as $rec_{c}$ is fixed, initially both *W* and η increase with an increase in $rec_{h}$, but then they decrease. Similarly, when $rec_{h}$ is fixed, both *W* and η exhibit an increasing trend followed by a decreasing one with respect to $rec_{c}$. This indicates the existence of an optimal region of relative entropy coherence for achieving the best performance of a heat engine. While moderate quantum coherence contributes to the enhancement of *W* and η to some extent, an excessive level of coherence may lead to the disappearance of *W* and η. Furthermore, unlike the cases depicted in Figure 6 and Figure 7, within the region of $rec_{h}\geq rec_{c}$ it is possible for the positive work *W* and efficiency η to exist. However, for the case of k=1.5, the heat engine can only output net work when $rec_{h}<rec_{c}$, as depicted in Figure 9. These observations imply that the relative strength between molecule−molecule interaction and field−molecule interaction plays a significant role in the QOHE.

## 4. Conclusions

We have studied the properties of QOHE by taking the dipole−dipole coupled polar molecules in an external electric field as the working substance. After numerically calculating, we have found that the work output and efficiency of the QOHE decrease monotonically with the increase in electric field strength under the conditions of $\epsilon_{h}=\epsilon_{c}$ and $\Omega_{h}\to \Omega_{c}\to \Omega_{h}$. However, the variation in efficiency with temperature differences between the cold and hot baths is non−monotonic. In the scenario where $\Omega_{h}=\Omega_{c}$ and $\epsilon_{h}\to \epsilon_{c}\to \epsilon_{h}$, there exist optimal values of Ω that can maximize the efficiency versus different temperatures $T_{c}$. For a given Ω, the efficiency is directly proportional to the temperature difference. Moreover, when $\epsilon_{c}/\epsilon_{h}=\Omega_{c}/\Omega_{h}=r$, it can be observed that both the work output and efficiency show an initial enhancement followed by a subsequent decrease with increasing *r*, ultimately reaching zero for r>1. For the case of $\epsilon_{c}/\epsilon_{h}=r$ with $\Omega_{c}$ = $\Omega_{h}$ = Ω, the work output and efficiency are found to be inversely proportional to Ω. Additionally, we have also discussed the effects of thermal entanglement and coherence on QOHE. It is found that the positive work can be output when $c_{h}<c_{c}$. Additionally, in the case of a relative coupling strength of k=1.5, it is not possible to generate positive work when $rec_{h}\geq rec_{c}$.

In summary, our results show that the polar molecule−based QOHE can be effectively manipulated by tuning the electric field and dipole−dipole interaction. As a contrast, by adjusting the magnetic field, coupling constant, and Dzyaloshinskii−Moriya interaction, the fine−tuning of work output and efficiency of the QOHE based on a three−qubit Heisenberg XXZ model can also be achieved [22]. It indicates that regulating the external field (e.g., electric field, magnetic field, and optical field) and the interactions between particles is a viable approach for improving the performance of the QHEs.

Although our results are theoretical and conceptual, they still hold significance for the diversification of QHE models and the understanding of quantum thermodynamics. Moreover, the QHEs in molecular systems may have potential applications in molecule-based quantum computing, such as powering quantum computers or serving as cooling agents to avoid thermal noises. We expect that our results could inspire the further studies on quantum computing and thermodynamics in polar molecule systems.

## Data Availability

The original contributions presented in the study are included in the article; further inquiries can be directed to the corresponding author.

## Abbreviations

The following abbreviations are used in this manuscript:

QHEs	quantum heat engines
QOHE	quantum Otto heat engine
